# Knowledge and Perceptions of Dentists Regarding E-Cigarettes: Implications for Oral Health and Public Awareness and Education

**DOI:** 10.3390/dj13030119

**Published:** 2025-03-10

**Authors:** Donika B. Shabani, Linda J. Dula, Zana Sllamniku Dalipi, Mirlinda S. Krasniqi, Aida Meto

**Affiliations:** 1 Department of Endodontics, University Dentistry Clinical Center of Kosovo, Medical Faculty, University of Prishtina, 10000 Prishtina, Kosovo; donika.bajrami@uni-pr.edu; 2 Department of Prosthetic, University Dentistry Clinical Center of Kosovo, Medical Faculty, University of Prishtina, 10000 Prishtina, Kosovo; 3 Department of Periodontology and Oral Medicine, University Dentistry Clinical Center of Kosovo, Medical Faculty, University of Prishtina, 10000 Prishtina, Kosovo; zana.sllamniku@uni-pr.edu (Z.S.D.); mirlinda.sopi@uni-pr.edu (M.S.K.); 4 Department of Dentistry, Faculty of Dental Sciences, University of Aldent, 1007 Tirana, Albania; 5 Department of Surgery, Medicine, Dentistry and Morphological Sciences with Interest in Transplant, Oncology and Regenerative Medicine, University of Modena and Reggio Emilia, 41125 Modena, Italy; 6 Department of Dental Research Cell, Dr. D. Y. Patil Dental College and Hospital, Dr. D.Y. Patil Vidyapeeth, Pimpri, Pune 411018, Maharashtra, India

**Keywords:** dentists’ knowledge, e-cigarettes, vaping, oral health, nicotine, periodontal disease, dental education, public health, smoking cessation, oral microbiome

## Abstract

**Background/Objectives:** The rising popularity of e-cigarette use is creating new challenges for oral healthcare. Factors such as targeted marketing, appealing flavors, and the perception that e-cigarettes are a safer alternative to traditional smoking have contributed to their widespread adoption. This trend is particularly prevalent among young adults and teenagers, many of whom turn to e-cigarettes despite having no prior history of regular cigarette use. This study aimed to evaluate dentists’ knowledge and perceptions regarding e-cigarettes, focusing on their health effects, regulatory status, and potential risks to oral health. Assessing dentists’ knowledge of e-cigarette risks is important, as they guide patients on the potential oral health dangers of vaping. E-cigarette use has been linked to several oral health problems. **Methods:** A cross-sectional survey was conducted among 189 dentists in Kosovo, comprising both general dentists and specialists. The questionnaire assessed participants’ awareness of e-cigarettes’ FDA approval status, their perceptions of health impacts, and their understanding of e-cigarettes’ association with oral conditions such as caries, periodontal disease, and oral cancer. Data were analyzed using SPSS 22, with significance set at *p* < 0.05. **Results:** The findings revealed significant knowledge gaps, with 74.1% of respondents being uncertain about the FDA approval status of e-cigarettes and 82.0% recognizing their harmful effects on general and oral health. Nearly half (49.7%) of the participants identified e-cigarettes as a potential risk factor for caries, while 66.1% and 64.6% associated their use with oral cancer and periodontal disease, respectively. Gender and professional specialization did not significantly influence these perceptions. **Conclusions:** Dentists need better education on the risks of e-cigarette use, including their harmful effects on oral health. Incorporating this knowledge into dental curricula and professional training is essential to give dentists the information needed to advise patients effectively. Raising public awareness through dental health professionals can help mitigate the oral health risks associated with e-cigarettes.

## 1. Introduction

Electronic cigarettes (e-cigarettes) were first invented in 1965 and introduced in North America in 2007. Since then, the market has grown, including over 400 brands and more than 7000 e-liquid flavors, some targeting younger users [1,2]. E-cigarettes have quickly gained popularity since their introduction, driven by aggressive marketing, widespread availability, and the perception that they are a safer alternative to traditional tobacco products. Initially marketed as a healthier option compared to smoking, e-cigarettes have continued to rise in popularity across a diverse range of users.

Commonly referred to as e-cigarettes, vaping devices, or vape pens, these devices are designed to deliver nicotine to users in the form of an aerosolized vapor. This vapor is created by heating a liquid, known as e-liquid or vape juice, which typically contains nicotine, flavorings, and other chemicals. While the term ’e-cigarettes’ is often used to describe all types of these devices, ’vaping devices’ is a broader term that includes e-cigarettes, mods, and pod systems. ’Vape pens’ specifically refer to portable, pen-like e-cigarettes, often used for vaping e-liquids or concentrates. Although most e-cigarettes are designed to deliver nicotine, not all of them do. Nicotine-free e-liquids are also available, giving users the option to vape without inhaling nicotine. This flexibility makes vaping devices suitable for various preferences, including those who want to enjoy vaping without the addictive substance [3,4]. E-cigarettes have become particularly popular among young people, who are drawn to the novelty, sensory experience, and recreational appeal they offer [5,6].

**Figure 1:**
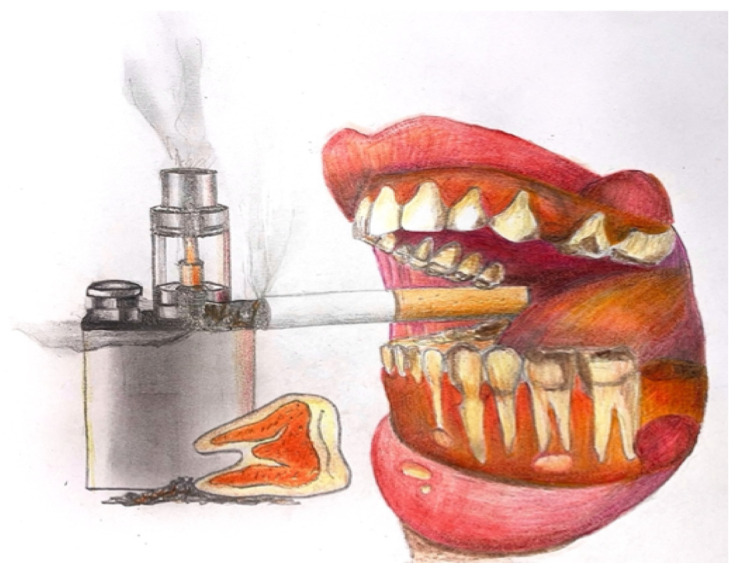
The image illustrates the effects of smoking and tobacco heating systems on oral health. It highlights the potential risks and long-term effects associated with these products, such as gum disease, tooth decay, and oral cancer. The figure also compares the impact of traditional smoking and tobacco heating systems, emphasizing the specific oral health issues linked to each. Illustration by Nensi Kallfani.

The World Health Organization (WHO) Study Group on Tobacco Product Regulation has recommended that e-cigarettes include proper labeling and health warnings due to concerns about their potential impact on health [7]. Despite their rising popularity, the long-term effects of e-cigarettes on overall and oral health are not fully known. Research suggests that e-cigarette use may be linked to gum diseases (gingivitis and periodontal inflammation), dry mouth (xerostomia), tooth decay, tooth staining, premature tooth loss, and a higher risk of oral cancer (Figure 1). However, many dental professionals are not fully aware of these risks, which could affect the quality of advice given to patients. Similarly to traditional smoking, e-cigarettes can contribute to periodontal issues because nicotine reduces blood flow to the gums [8]. In this context, it is crucial to understand the extent of dentists’ knowledge about e-cigarettes and their impact on oral health.

The potential short-term risks of e-cigarette use on oral health underscore the need for greater awareness among healthcare professionals and the public [8,9,10,11].

As the popularity of e-cigarettes continues to grow, many users are still unaware of the potential health risks associated with their use. Therefore, health professionals need to stay informed about the latest research on e-cigarettes. This includes understanding their components, the ingredients in refill liquids, and the health risks associated with their use [12,13].

Dentists play a crucial role in raising awareness about e-cigarettes to promote better oral health. Training for healthcare professionals should ideally include a comprehensive understanding of e-cigarettes and their components, the chemical composition of e-liquids, associated health risks, and their effectiveness as a smoking cessation tool. With this knowledge, dentists and other providers can offer informed guidance, address patient concerns, and contribute to health promotion and disease prevention [14,15].

This study aimed to evaluate dentists’ level of knowledge regarding the use of e-cigarettes and their effects on oral health. The objectives were to assess the importance of this knowledge for dental practice and to examine dentists’ self-perception of their ability to guide patients about e-cigarette use. The study highlights the need for continuing education, recommending that dental professionals participate in workshops and seminars focused on tobacco cessation and the impacts of vaping on both oral and general health.

## 2. Materials and Methods

### 2.1. Study Design and Participants

Although the total number of dentists in Kosovo is significantly higher, this study selected a sample of 189 licensed dentists from the Dental Chamber of Kosovo, including both general dentists and specialists in prosthetics, oral medicine, dental pathology, oral surgery, and orthodontics. Participants worked in both public and private healthcare institutions. To ensure a diverse and representative sample, the study included dentists from various regions of Kosovo. A random sampling approach was used to select participants, with inclusion dependent on their availability and readiness to participate during the study period. Only actively practicing dentists with professional experience were included to maintain the reliability and relevance of responses.

The sample size of 189 dentists was determined to achieve a 95% confidence level with a margin of error of ±7.13%, ensuring a representative sample of active dental practitioners in Kosovo.

No specific exclusion criteria were applied. The questionnaire was distributed in printed form to all the dentists who completed and signed it. The study was conducted between July and August 2024.

The questions were neutrally formulated to avoid any potential bias, ensuring honest responses and enhancing the study’s objectivity and results reliability. The confidentiality of the responses was ensured through specific measures to protect privacy. All data were collected anonymously, without identifying the individuals who participated in the study. Participants were informed in advance about the anonymous nature of the survey and that no personal information would be linked to their responses. The data were stored in secure systems, with access granted only to the study authors for analysis purposes. Additionally, the responses were coded to ensure that no individual information could be identified, thus enhancing the transparency and reliability of the study.

### 2.2. Ethical Considerations

The study objectives were explained to all participants, and informed consent was obtained. Participation was voluntary, and the participants were instructed to complete the survey only once. No personal identifiers were collected to ensure anonymity. Ethical approval for this study was granted by the Research Ethics Committee of the Dental Chamber of Kosovo under approval number No: 48-1-30.05.2024.

### 2.3. Survey Instrument

A structured questionnaire was specifically developed for this study to assess dentists’ knowledge, perceptions, and usage patterns of e-cigarettes, as well as their awareness of health risks and oral health implications. It consisted of four sections covering participant demographics, e-cigarette usage, health awareness, and attitudes toward their impact on oral health.

To ensure validity, the questionnaire was pilot-tested with 30 randomly selected dentists from Kosovo. Their feedback was used to refine the clarity, accuracy, and relevance of the questions. Necessary modifications were made to enhance the precision of the questions and their ability to capture meaningful insights. The reliability of the questionnaire was confirmed by evaluating the consistency of responses across the pilot group, ensuring its reproducibility and internal coherence before inclusion in the final survey.

This validation process was crucial in developing a robust assessment tool that accurately measures dentists’ understanding and attitudes toward e-cigarettes. The refined questionnaire serves as a valuable instrument for informing public health strategies and educational initiatives aimed at both dental professionals and the general public regarding the risks associated with e-cigarette use.

#### 2.3.1. Participant Demographics

The first section collected basic demographic and professional information, such as the participants’ gender, professional background, and level of education. These data were used to categorize respondents and analyze findings based on these factors.

#### 2.3.2. Analysis of Dentists’ E-Cigarette and Tobacco Usage Patterns

The second section analyzed the participants’ knowledge and usage patterns of e-cigarettes and tobacco. Key aspects included the following:

- Identifying the percentage of dentists who use e-cigarettes or tobacco.
- Determining the frequency and duration of e-cigarette use among dentists.
- Comparing usage patterns based on gender, specialization, and years of practice.

#### 2.3.3. Knowledge of the Health Effects of E-Cigarettes

The third section evaluated the participants’ understanding of the general health implications of e-cigarette use. Key points included the following:

- Assessing knowledge of the overall health risks and benefits associated with e-cigarettes.
- Evaluating awareness of regulatory approval, such as FDA certification, and perceptions of their safety.

This section contained 10 statements with three response options: “Yes”, “No”, and “I don’t know”.

#### 2.3.4. Knowledge of Oral Health Impacts of E-Cigarettes

The fourth section focused on the participants’ knowledge of how e-cigarettes impact oral health. Key points included the following:

- Understanding the association between e-cigarettes and oral health issues, such as caries, periodontal disease, xerostomia, and mucosal lesions.
- Assessing the impact of e-cigarettes on the oral microbiome and related disorders.
- Comparing the long-term effects of e-cigarettes on oral health to those of traditional tobacco products.

This section included eight statements based on the existing literature [16,17,18,19], with response options of “Yes”, “No”, and “I don’t know”.

### 2.4. Data Analysis

The data collected from the questionnaires were analyzed using the Statistical Package for the Social Sciences (SPSS) version 22.0. Descriptive statistics (frequencies and percentages) were used to summarize the data. All analyses were stratified by both sex and professional training to ensure a comprehensive assessment of differences between groups. The Chi-square test (χ^2^) was used to assess associations between categorical variables, and Fisher’s Exact Test was applied when expected counts were below 5.

The analysis focused on assessing the relationship between dentists’ knowledge and e-cigarette use using descriptive statistics and association tests. No further adjustments for confounders or multivariable analyses were conducted.

Statistical significance was set at *p* < 0.05 for all tests. The classification of knowledge levels was based on responses to multiple knowledge-related questions, but no specific categorization or regression analysis was performed to quantify its association with e-cigarette use.

## 3. Results

### 3.1. General Characteristics of Participants

The study included a total of 189 dentists, comprising 103 general dentists (54.5%) and 86 specialists (45.5%). Among the participants, 108 (57.1%) were female, and 81 (42.9%) were male.

### 3.2. Use of E-Cigarettes by Gender

The use of e-cigarettes and tobacco was analyzed by gender, as shown in Table 1. Among the 189 participants, 46.0% reported using either e-cigarettes or tobacco, with a significantly higher prevalence among males (59.3%) compared to females (36.1%) (*p* = 0.003). Additionally, 43.9% of participants reported that a family member used e-cigarettes, with no significant difference between genders (*p* = 0.783).

Exclusive e-cigarette use was reported by 9.5% of participants (10.2% females vs. 8.6% males), while males were more likely to use only tobacco (33.3% vs. 14.8% in females; *p* = 0.026). Among e-cigarette users, 14.3% reported using them for more than two years.

### 3.3. Use of E-Cigarettes by Professional Training

The study analyzed e-cigarette and tobacco use according to professional training, comparing general dentists and specialists. As shown in Table 2, 45.6% of general dentists and 46.5% of specialists reported having used either e-cigarettes or tobacco, with no statistically significant difference (*p* = 0.904).

Among general dentists, 78.6% reported never using e-cigarettes, compared to 73.3% of specialists. A slightly higher proportion of specialists (17.4%) reported using e-cigarettes for more than two years compared to general dentists (11.7%), though this difference was not statistically significant (*p* = 0.690).

### 3.4. Knowledge and Perceptions of E-Cigarette Safety, Health Effects, and Usage Implications

This study assessed the participants’ knowledge and perceptions regarding the safety, health effects, and implications of e-cigarette use, as summarized in Table 3. A majority of participants (74.1%) were unsure whether e-cigarettes are FDA-approved for safe consumption, with only 3.7% affirming that they are approved. A significantly higher percentage of males (7.4%) compared to females (0.9%) believed in FDA approval (*p* = 0.047). Regarding the perception of harm, 82.0% of participants agreed that e-cigarettes are harmful to general and oral health, while only 1.6% believed otherwise, and 16.4% remained uncertain. No significant gender differences were observed (*p* = 0.373).

Opinions were divided on whether e-cigarettes do not affect passive smoking, with 22.2% of participants agreeing, 22.8% disagreeing, and 55.0% being unsure. Similarly, when comparing e-cigarettes to traditional tobacco products, 19.6% believed that e-cigarettes are less harmful, 37.6% disagreed, and 42.9% expressed uncertainty. In both cases, no significant gender differences were identified (*p* = 0.485 and *p* = 0.253, respectively).

When asked if e-cigarettes help relieve daily stress, 17.5% of participants agreed, 32.8% disagreed, and 49.7% were uncertain, with no significant differences between genders (*p* = 0.944). Nearly half of the participants (48.7%) agreed that e-cigarettes create addiction, 10.6% disagreed, and 40.7% were unsure, with no significant gender differences observed (*p* = 0.487). In terms of carcinogenic effects, 16.4% of participants believed that e-cigarettes are less carcinogenic than tobacco cigarettes, 32.8% disagreed, and 50.8% were unsure, with no significant gender differences (*p* = 0.398).

The perspectives on whether e-cigarettes aid in smoking cessation varied, with 22.2% of participants agreeing, 42.9% disagreeing, and 34.9% expressing uncertainty, with no significant gender differences observed (*p* = 0.197). Finally, regarding knowledge of the composition of e-cigarettes, 31.7% of participants reported being aware of the composition, 30.2% were not, and 38.1% were unsure, with no significant gender differences observed (*p* = 0.737).

### 3.5. Knowledge and Perceptions of E-Cigarettes by Professional Training

This study examined the knowledge and perceptions of e-cigarettes among general dentists and specialists, as presented in Table 4. A large majority of both general dentists (77.7%) and specialists (69.8%) were unsure whether e-cigarettes are FDA-approved for safe consumption, while only 2.9% and 4.7%, respectively, affirmed FDA approval. No statistically significant difference was found between the groups (*p* = 0.454).

Regarding the harmfulness of e-cigarettes, 81.6% of general dentists and 82.6% of specialists agreed that they are harmful to general and oral health, with no significant difference (*p* = 0.702). Similarly, uncertainty was evident in the participants’ perceptions of e-cigarettes’ effects on passive smoking, with 60.2% of general dentists and 48.8% of specialists reporting that they did not know, and no significant difference between the groups (*p* = 0.078).

When comparing the harm of e-cigarettes to traditional tobacco, 19.4% of general dentists and 19.8% of specialists believed that e-cigarettes were less harmful, while a comparable proportion (36.9% and 38.4%, respectively) disagreed. The majority remained uncertain, with no significant difference observed (*p* = 0.967).

Regarding stress relief, 13.6% of general dentists and 22.1% of specialists agreed that e-cigarettes help relieve daily stress, while the majority were unsure (*p* = 0.228). Regarding the addictive potential of e-cigarettes, 53.4% of general dentists and 43.0% of specialists agreed that they are addictive, while 12.6% and 8.1%, respectively, disagreed (*p* = 0.107).

In terms of carcinogenicity, 16.5% of general dentists and 16.3% of specialists believed e-cigarettes were less carcinogenic than traditional tobacco, while 29.1% and 37.2%, respectively, disagreed. Most participants remained uncertain, with no significant differences (*p* = 0.471).

When asked whether e-cigarettes help with smoking cessation, 27.2% of general dentists and 16.3% of specialists agreed, while 42.7% and 43.0%, respectively, disagreed, with the remainder being unsure (*p* = 0.134). Knowledge of the composition of e-cigarettes was slightly higher among specialists (36.0%) than general dentists (28.2%), though the difference was not significant (*p* = 0.458).

Overall, the findings reveal similar levels of knowledge and perceptions between general dentists and specialists, with significant uncertainty across both groups about e-cigarettes’ safety, health effects, and their role in smoking cessation.

### 3.6. Perceptions of the Oral Health Effects of E-Cigarettes by Gender

This study examined the participants’ perceptions of how e-cigarettes affect oral health, with results presented in Table 5. Regarding the impact of e-cigarettes on caries incidence, 49.7% of participants agreed that e-cigarettes increase the risk of caries, with slightly more females (50.9%) than males (48.1%) affirming this. However, 11.1% believed that there was no effect, and 39.2% were uncertain, with no significant gender difference (*p* = 0.166).

When asked whether e-cigarettes affect the oral cavity, 75.1% of participants agreed, with nearly identical responses from females (75.0%) and males (75.3%). A small percentage (2.6%) believed that there was no effect, while 22.2% were unsure (*p* = 0.991). Similar responses were observed for xerostomia, mouth burning, and halitosis, with 70.4% agreeing that e-cigarettes had an impact, 1.6% disagreeing, and 28.0% being unsure (*p* = 0.629).

In terms of cancer risk, 66.1% of participants believed that e-cigarettes increase the risk of oral cancer through cytotoxic, genotoxic, and oncogenic effects. Only 0.5% disagreed, and 33.3% were unsure, with no significant gender difference (*p* = 0.773). Regarding the prevalence of periodontal lesions, 64.6% of participants agreed that e-cigarettes have an impact, 7.9% disagreed, and 27.5% were unsure, again with no significant gender difference (*p* = 0.403).

For other oral conditions such as lingua nigra villosa, nicotinic stomatitis, and tongue discolorations, 61.4% of participants agreed that e-cigarettes could cause these effects, 3.2% disagreed, and 35.4% were uncertain (*p* = 0.812). When asked about the impact on teeth, including discoloration, fractures, pain, and increased cariogenic bacteria, 64.0% of participants agreed, 6.9% disagreed, and 29.1% were uncertain (*p* = 0.720).

Lastly, the answers regarding perceptions of e-cigarettes’ impact on the oral microbiome showed that 65.1% agreed, 4.2% disagreed, and 30.7% were uncertain (*p* = 0.793). Across all these areas, no significant gender differences were identified.

### 3.7. Perceptions of the Oral Health Effects of E-Cigarettes by Professional Training

This study assessed the participants’ perceptions of the oral health effects of e-cigarettes based on their professional training as general dentists or specialists, as shown in Table 6. Regarding the effect of e-cigarettes on caries incidence, 54.4% of general dentists and 44.2% of specialists agreed that e-cigarettes increase caries risk, while 10.7% of general dentists and 11.6% of specialists believed that there was no effect. The remaining participants were uncertain, with no significant difference observed between the two groups (*p* = 0.361).

When asked if e-cigarettes affect the oral cavity, 76.7% of general dentists and 73.3% of specialists agreed, while a very small proportion (1.0% and 4.7%, respectively) disagreed. Approximately 22% of both groups were uncertain, and no significant difference was found (*p* = 0.290). A similar trend was observed for the effect of e-cigarettes on xerostomia, mouth burning, and halitosis, with 71.8% of general dentists and 68.6% of specialists agreeing, 3.5% of specialists disagreeing, and the remaining participants being unsure (*p* = 0.745).

In terms of the potential for e-cigarettes to increase the risk of oral cancer, 68.0% of general dentists and 64.0% of specialists agreed, while only 1.2% of specialists disagreed. A notable proportion of participants were uncertain (32.0% of general dentists and 34.9% of specialists), but no significant difference was observed (*p* = 0.670).

Regarding the prevalence of periodontal lesions, 64.1% of general dentists and 65.1% of specialists believed that e-cigarettes have an effect, while 9.7% of general dentists and 5.8% of specialists disagreed. Approximately 26–29% of participants were unsure, with no significant difference between the groups (*p* = 0.594). For other conditions such as lingua nigra villosa, nicotinic stomatitis, and tongue discolorations, 65.0% of general dentists and 57.0% of specialists agreed, while a small proportion (1.9% and 4.7%, respectively) disagreed. Uncertainty remained high, with 33.0% of general dentists and 38.4% of specialists expressing doubts (*p* = 0.375).

When asked whether e-cigarettes affect teeth (e.g., discoloration, pain, fractures, and increased cariogenic bacteria), 67.0% of general dentists and 60.5% of specialists agreed, while 6.8% and 7.0%, respectively, disagreed, and the remainder were unsure (*p* = 0.618). Finally, regarding the impact of e-cigarettes on disorders of the oral microbiome, 69.9% of general dentists and 59.3% of specialists agreed, while 2.9% and 5.8%, respectively, disagreed. A substantial proportion of both groups were uncertain, with no significant difference between them (*p* = 0.266).

## 4. Discussion

The increasing prevalence of e-cigarette use highlights the need for dental professionals to understand their potential impact on oral health and provide informed guidance to patients [20]. This study explored dentists’ knowledge and perceptions of e-cigarettes, focusing on their associated risks, regulatory status, and the dentists’ ability to communicate this information effectively. The WHO offers several key recommendations concerning e-cigarettes and their impact on oral and public health. These recommendations may include the following:

1. **Dissemination of Health Risk Information:** The WHO emphasizes the importance of educating the public and healthcare professionals about the health risks of e-cigarette use, including potential harmful effects on oral health, such as damage to gum tissue and an increased risk of oral diseases.
2. **Regulation of E-Cigarette Sales and Use:** The WHO recommends that countries enforce strict regulations on the sale and use of e-cigarettes, including banning their sale to minors and prohibiting their use in public spaces.
3. **Further Research and Studies:** The WHO encourages continued research to gain a better understanding of the long-term effects of e-cigarettes on both oral and general health, including studies on the impact of different components in e-cigarette liquids.
4. **Support for Healthcare Professionals:** The WHO underscores the importance of training dentists and other healthcare professionals to recognize and address the oral health consequences of e-cigarette use, ensuring that they stay informed about risks and treatment recommendations.
5. **Public Education:** The WHO supports initiatives to educate the public about e-cigarettes and their potential risks, emphasizing that they are not a risk-free alternative to conventional tobacco.

The findings highlight variations in dentists’ knowledge levels regarding e-cigarettes and their potential impact on usage patterns. However, the method used to classify knowledge levels was not explicitly defined, and no adjusted analysis was conducted to establish a direct relationship between knowledge and e-cigarette use. These results underscore the need for enhanced education and training to improve awareness of e-cigarette risks and inform professional behavior.

Despite the observed association, a substantial proportion of dentists across all knowledge levels reported using e-cigarettes, indicating that knowledge alone is not the sole determinant of behavior. Various factors, including stress relief, social norms, exposure to marketing, and personal smoking history, may also play a significant role in influencing e-cigarette use among dental professionals.

One possible explanation for this trend is that dentists with higher knowledge levels may feel a stronger professional obligation to adhere to health standards, making them more cautious about engaging in behaviors that contradict public health recommendations. However, the continued use of e-cigarettes among well-informed professionals suggests that behavioral, psychological, and social influences may, sometimes, exert greater influence than knowledge-driven choices.

This survey identified key trends in e-cigarette and tobacco use, highlighting notable gender differences. Approximately 46.0% of respondents reported using e-cigarettes or tobacco, with a higher prevalence among males (59.3%) compared to females (36.1%). Additionally, 43.9% of participants reported having family members who use e-cigarettes, emphasizing the role of social acceptance and peer influence in normalizing their use [21]. Exclusive e-cigarette use was low (9.5%), with females more likely to use both e-cigarettes and tobacco, while males predominantly used tobacco alone (*p* < 0.05). These findings underscore the influence of social and cultural factors on e-cigarette use.

Among e-cigarette users, 14.3% had been using them for more than two years, often multiple times daily. This aligns with findings that e-cigarettes are perceived as addictive, comparable to alcohol, and more so than marijuana [22,23]. The relatively recent adoption of e-cigarettes highlights the importance of monitoring their long-term effects and patterns of use [23].

This study uncovered significant knowledge gaps regarding FDA approval, with 74.1% of respondents uncertain about whether e-cigarettes are FDA-approved for safe consumption. Female dentists (25.0%) were more likely than males (18.5%) to believe e-cigarettes are not FDA-approved (*p* < 0.05). Public health strategies should focus on promoting FDA-approved smoking cessation methods, which remain the most reliable option for quitting smoking [24,25]. E-cigarettes, which lack robust evidence supporting their safety and effectiveness, should not be promoted as an alternative to approved treatments. The introduction of FDA regulations in 2016 requiring ingredient labeling has improved consumer awareness, but further oversight is necessary to ensure safety [26].

Health impacts were another significant concern, with 82.0% of respondents acknowledging the harmful effects of e-cigarettes on general and oral health. Female respondents expressed slightly higher concern (85.2%) than males (77.8%), though this difference was not statistically significant. These findings align with research suggesting that e-cigarettes contribute to oral health issues such as xerostomia, halitosis, and increased caries risk. For example, Irusa et al. [27] linked vaping to an increased risk of tooth decay, exacerbated by dry mouth.

Concerns about oral cancer risk and periodontal disease were also prominent, with 66.1% and 64.6% of respondents, respectively, acknowledging potential impacts. Studies by Jeong et al. [28] and Xu et al. [29] support these findings, linking vaping to periodontal damage and adverse effects on oral biofilms. Additionally, Ramenzoni et al. [30] highlighted the cytotoxic and inflammatory effects of vaping on oral tissues, further underscoring the risks.

Uncertainty persists regarding the relative safety of e-cigarettes compared to traditional tobacco. While 19.6% of respondents believed that e-cigarettes are less harmful than tobacco, 37.6% disagreed, and 42.9% were unsure. Similarly, only 22.2% of participants considered e-cigarettes effective for smoking cessation, with 42.9% disagreeing and 34.9% being uncertain. These results align with prior studies indicating that the long-term safety and efficacy of e-cigarettes remain poorly understood [31,32]. The lack of conclusive evidence warrants a cautious approach, emphasizing the need for further research to fully understand the implications of e-cigarette use [32,33].

This study also revealed low awareness of the composition of e-cigarettes, with 38.1% of respondents being unsure about their contents. This lack of knowledge is concerning, as flavored vaping liquids have been shown to negatively impact biofilm formation and bacterial proliferation in the oral microbiome [32]. Over 60% of respondents expressed concern about conditions such as black hairy tongue, tooth discoloration, and oral microbiome disorders, reflecting the growing awareness of these risks.

Emerging research highlights the potential of salivary biomarkers, such as microRNAs, in detecting oral health issues associated with tobacco and alternative smoking devices. Increased levels of pro-inflammatory cytokines, such as IL-1β and TNF-α, have been observed in the saliva of e-cigarette users compared to non-smokers. These cytokines are associated with inflammation and may contribute to the development of periodontal disease [34]. Another study [35] demonstrated that salivary microRNAs could serve as innovative biomarkers for the early diagnosis of oral diseases, comparing conventional cigarette smokers with users of tobacco heating systems. Their findings revealed significant differences in the expression of salivary microRNAs between the two groups, indicating that alternative smoking devices, such as tobacco heating systems, also pose risks to oral health. These results align with our study’s findings, which emphasize the adverse effects of e-cigarette use on oral health, including increased risks of periodontal disease, xerostomia, and the disruption of the oral microbiome. Incorporating salivary biomarkers as diagnostic tools in future research could provide valuable insights into the biological impact of e-cigarettes and similar products, enabling earlier detection and intervention for vaping-related oral conditions.

This study has several limitations. While the sample size was adequate for an exploratory analysis, it may not fully capture the range of perspectives among dental professionals. The reliance on self-reported data introduces the potential for recall and social desirability biases, which could affect response accuracy. Additionally, the study’s focus on Kosovo may limit the applicability of findings to regions with different regulatory frameworks and cultural contexts. The cross-sectional design further restricts the ability to assess changes in knowledge and perceptions over time.

Future research with a larger and more diverse sample is recommended to enhance statistical precision and confirm observed trends. Longitudinal studies should be conducted to track changes in perceptions and knowledge over time. Educational interventions must address gaps in awareness regarding FDA regulations, health risks, and the role of e-cigarettes in smoking cessation. Collaboration with dental associations and regulatory bodies will be crucial for effectively disseminating evidence-based information. Further research on the long-term effects of e-cigarette use, particularly its impact on oral and general health, is essential to guide clinical practice and public health policies.

Beyond knowledge dissemination, educational programs should integrate behavior-change strategies to enhance smoking cessation counseling and empower dental professionals as role models for their patients. Addressing existing knowledge gaps through targeted education and evidence-based guidance will enable dentists to provide informed patient counseling, mitigate the risks associated with e-cigarette use, and contribute to better oral health outcomes.

Through continuous education, dental professionals can improve their ability to address the challenges associated with e-cigarette use, leading to better oral health outcomes for patients. Implementing educational campaigns for both patients and dental professionals is crucial to reference the growing concerns about e-cigarettes and their impact on oral health.

Public policies can facilitate the integration of smoking and vaping cessation programs into dental practices. By providing dental professionals with the necessary tools and knowledge, these programs can effectively help patients quit, leading to improved overall oral health outcomes.

## 5. Conclusions

This study highlights the need for greater awareness and education among dental professionals regarding e-cigarettes and their potential impact on oral and general health. While most participants recognized their harmful effects, significant knowledge gaps were identified, particularly regarding FDA approval, composition, and their role in smoking cessation. Findings also reveal uncertainty about the long-term safety and effectiveness of e-cigarettes despite their common perception as a safer alternative to traditional tobacco products. Their potential risks, including caries, xerostomia, periodontal disease, and carcinogenic effects, warrant careful consideration in dental practice. To provide accurate patient guidance, dental professionals must be equipped with evidence-based knowledge of the risks and benefits of e-cigarettes. Public policies regulating e-cigarette use should also be integrated into dental care protocols to ensure consistent patient counseling. Addressing these knowledge gaps requires targeted educational initiatives, such as workshops, seminars, and collaborations with dental associations, to provide up-to-date information on health risks, regulations, and best practices for patient counseling. Strengthening education will empower dental professionals to support informed decision-making and contribute to better oral health outcomes.

## Figures and Tables

**Table 1:** Use of e-cigarettes by gender.

	Female		Male		Total		*p*-Value
	N	%	N	%	N	%	
Total	108	100.0	81	100.0	189	100.0	
**Have you ever used e-cigarettes or tobacco?**							
Yes	39	36.1	48	59.3	87	46.0	*p* = 0.003
No	69	63.9	33	40.7	102	54.0	
**Does any member of your family use e-cigarettes?**							
Yes	46	42.6	37	45.7	83	43.9	*p* = 0.783
No	62	57.4	44	54.3	106	56.1	
**Do you use tobacco or e-cigarettes?**							
E-cigarettes	11	10.2	7	8.6	18	9.5	* *p* = 0.026
None *	74	68.5	44	54.3	118	62.4	
Both	7	6.5	3	3.7	10	5.3	
Only tobacco	16	14.8	27	33.3	43	22.8	
**How long have you been using e-cigarettes?**							
Did not use it	84	77.8	60	74.1	144	76.2	*p* = 0.814
1–2 months	3	2.8	-	-	3	1.6	
1–2 years	7	6.5	8	9.9	15	7.9	
>2 years	14	13.0	13	16.0	27	14.3	
**Approximately, how much do you consume e-cigarettes during the day?**							
<20 times a day	4	3.7	6	7.4	10	5.3	*p* = 0.267
≥20 times a day	2	1.9	4	4.9	6	3.2	
Did not use it	88	81.5	65	80.2	153	81.0	
A few times a day	14	13.0	6	7.4	20	10.6	

The statistical test used for comparisons in this table was the Chi-square test (χ^2^).

**Table 2:** Use of e-cigarettes according to education/professional training.

	Dentist		Specialist		*p*-Value
	N	%	N	%	
Total	103	100.0	86	100.0	
**Gender**					
F	53	51.5	55	64.0	*p* = 0.114
M	50	48.5	31	36.0	
**Have you ever used e-cigarettes or tobacco?**					
Yes	47	45.6	40	46.5	*p* = 0.904
No	56	54.4	46	53.5	
**Does any member of your family use e-cigarettes?**					
Yes	47	45.6	36	41.9	*p* = 0.709
No	56	54.4	50	58.1	
**Do you use tobacco or e-cigarettes?**					
E- cigarettes	8	7.8	10	11.6	*p* = 0.629
Not anyone	64	62.1	54	62.8	
Both	7	6.8	3	3.5	
Only tobacco	24	23.3	19	22.1	
**How long have you been using e-cigarettes?**					
Did not use it	81	78.6	63	73.3	*p* = 0.690
1–2 months	2	1.9	1	1.2	
1–2 years	8	7.8	7	8.1	
>2 years	12	11.7	15	17.4	
**Approximately, how much do you consume e-cigarettes during the day?**					
Did not use it	84	81.6	69	80.2	*p* = 0.217
A few a day	11	10.7	9	10.5	
<20 times a day	3	2.9	7	8.1	
≥20 times a day	5	4.9	1	1.2	

The statistical test used for comparisons in this table was the Chi-square test (χ^2^). No significant differences were found between dentists and specialists for any of the categories presented (*p* > 0.05 for all comparisons).

**Table 3:** Knowledge and perceptions of e-cigarette safety, health effects, and usage implications.

	Female		Male		Total		*p*-Value
	N	%	N	%	N	%	
Total	108	100.0	81	100.0	189	100.0	
**Are e-cigarettes FDA-approved as safe for consumption?**							
Yes *	1	0.9	6	7.4	7	3.7	* *p* = 0.047
No	27	25.0	15	18.5	42	22.2	
I don’t know	80	74.1	60	74.1	140	74.1	
**Are e-cigarettes harmful to general and oral health?**							
Yes	92	85.2	63	77.8	155	82.0	*p* = 0.373
No	1	0.9	2	2.5	3	1.6	
I don’t know	15	13.9	16	19.8	31	16.4	
**Does the use of e-cigarettes have no effect on passive smoking?**							
Yes	23	21.3	19	23.5	42	22.2	*p* = 0.485
No	28	25.9	15	18.5	43	22.8	
I don’t know	57	52.8	47	58.0	104	55.0	
**Are e-cigarettes less harmful than tobacco cigarettes?**							
Yes	19	17.6	18	22.2	37	19.6	*p* = 0.253
No	46	42.6	25	30.9	71	37.6	
I don’t know	43	39.8	38	46.9	81	42.9	
**Do e-cigarettes help to relieve daily stress?**							
Yes	18	16.7	15	18.5	33	17.5	*p* = 0.944
No	36	33.3	26	32.1	62	32.8	
I don’t know	54	50.0	40	49.4	94	49.7	
**Although there are not many long-term studies on the effects of e-cigarettes, do they have fewer side effects on patients than tobacco cigarettes?**							
Yes	21	19.4	21	25.9	42	22.2	*p* = 0.325
No	32	29.6	17	21.0	49	25.9	
I don’t know	55	50.9	43	53.1	98	51.9	
**Do e-cigarettes create addiction?**							
Yes	56	51.9	36	44.4	92	48.7	*p* = 0.487
No	12	11.1	8	9.9	20	10.6	
I don’t know	40	37.0	37	45.7	77	40.7	
**Are e-cigarettes less carcinogenic than tobacco cigarettes?**							
Yes	15	13.9	16	19.8	31	16.4	*p* = 0.398
No	39	36.1	23	28.4	62	32.8	
I don’t know	54	50.0	42	51.9	96	50.8	
**Do you believe that e-cigarettes help to stop or reduce smoking?**							
Yes	19	17.6	23	28.4	42	22.2	*p* = 0.197
No	48	44.4	33	40.7	81	42.9	
I don’t know	41	38.0	25	30.9	66	34.9	
**Do you know the composition of electronic cigarettes?**							
Yes	33	30.6	27	33.3	60	31.7	*p* = 0.737
No	35	32.4	22	27.2	57	30.2	
I don’t know	40	37.0	32	39.5	72	38.1	

The statistical test used for comparisons in this table was the Chi-square test (χ^2^).

**Table 4:** Level of knowledge of dentists about cigarettes according to professional education/training.

	Dentist		Specialist		*p*-Value
	N	%	N	%	
Total	103	100.0	86	100.0	
**Are e-cigarettes FDA-approved as safe for consumption?**					
Yes	3	2.9	4	4.7	*p* = 0.454
No	20	19.4	22	25.6	
I don’t know	80	77.7	60	69.8	
**Are e-cigarettes harmful to general and oral health?**					
Yes	84	81.6	71	82.6	*p* = 0.702
No	1	1.0	2	2.3	
I don’t know	18	17.5	13	15.1	
**Does the use of e-cigarettes have no effect on passive smoking?**					
Yes	24	23.3	18	20.9	*p* = 0.078
No	17	16.5	26	30.2	
I don’t know	62	60.2	42	48.8	
**Are e-cigarettes less harmful than tobacco cigarettes?**					
Yes	20	19.4	17	19.8	*p* = 0.967
No	38	36.9	33	38.4	
I don’t know	45	43.7	36	41.9	
**Do e-cigarettes help to relieve daily stress?**					
Yes	14	13.6	19	22.1	*p* = 0.228
No	33	32.0	29	33.7	
I don’t know	56	54.4	38	44.2	
**Although there are not many long-term studies on the effects of e-cigarettes, do they have fewer side effects on patients than tobacco cigarettes?**					
Yes	24	23.3	18	20.9	*p* = 0.078
No	20	19.4	29	33.7	
I don’t know	59	57.3	39	45.3	
**Do e-cigarettes create addiction?**					
Yes	55	53.4	37	43.0	*p* = 0.107
No	13	12.6	7	8.1	
I don’t know	35	34.0	42	48.8	
**Are e-cigarettes less carcinogenic than tobacco cigarettes?**					
Yes	17	16.5	14	16.3	*p* = 0.471
No	30	29.1	32	37.2	
I don’t know	56	54.4	40	46.5	
**Do you believe that e-cigarettes help to stop or reduce smoking?**					
Yes	28	27.2	14	16.3	*p* = 0.134
No	44	42.7	37	43.0	
I don’t know	31	30.1	35	40.7	
**Do you know the composition of electronic cigarettes?**					
Yes	29	28.2	31	36.0	*p* = 0.458
No	34	33.0	23	26.7	
I don’t know	40	38.8	32	37.2	

The statistical test used for comparisons in this table was the Chi-square test (χ^2^). No significant differences were found between dentists and specialists for any of the categories presented (*p* > 0.05 for all comparisons).

**Table 5:** Level of knowledge about the impact of e-cigarettes on oral health by gender.

	Female		Male		Total		*p*-Value
	N	%	N	%	N	%	
Total	108	100.0	81	100.0	189	100.0	
**Do e-cigarettes affect the increase in the incidence of caries?**							
Yes	55	50.9	39	48.1	94	49.7	*p* = 0.166
No	8	7.4	13	16.0	21	11.1	
I don’t know	45	41.7	29	35.8	74	39.2	
**Does the use of e-cigarettes affect the oral cavity?**							
Yes	81	75.0	61	75.3	142	75.1	*p* = 0.991
No	3	2.8	2	2.5	5	2.6	
I don’t know	24	22.2	18	22.2	42	22.2	
**Do e-cigarettes affect xerostomia, mouth burning, and halitosis?**							
Yes	74	68.5	59	72.8	133	70.4	*p* = 0.629
No	3	2.8	-	-	3	1.6	
I don’t know	31	28.7	22	27.2	53	28.0	
**Do e-cigarettes increase the risk of oral cancer (cytotoxic, genotoxic, and oncogenic effects)?**							
Yes	70	64.8	55	67.9	125	66.1	*p* = 0.773
No	1	0.9	-	-	1	0.5	
I don’t know	37	34.3	26	32.1	63	33.3	
**Do e-cigarettes affect the prevalence of periodontal lesions?**							
Yes	72	66.7	50	61.7	122	64.6	*p* = 0.403
No	10	9.3	5	6.2	15	7.9	
I don’t know	26	24.1	26	32.1	52	27.5	
**Do e-cigarettes affect lingua nigra villosa, nicotinic stomatitis, and other tongue discolorations?**							
Yes	65	60.2	51	63.0	116	61.4	*p* = 0.812
No	6	5.6	-	-	6	3.2	
I don’t know	37	34.3	30	37.0	67	35.4	
**Do e-cigarettes affect teeth (tooth discoloration, pain, fractures, and increase in cariogenic bacteria)?**							
Yes	71	65.7	50	61.7	121	64.0	*p* = 0.720
No	8	7.4	5	6.2	13	6.9	
I don’t know	29	26.9	26	32.1	55	29.1	
**Do e-cigarettes affect disorders of the oral microbiome?**							
Yes	69	63.9	54	66.7	123	65.1	*p* = 0.793
No	4	3.7	4	4.9	8	4.2	
I don’t know	35	32.4	23	28.4	58	30.7	

The statistical test used for comparisons in this table was the Chi-square test (χ^2^). No significant differences were found between females and males for any of the categories presented (*p* > 0.05 for all comparisons).

**Table 6:** Level of knowledge about the impact of e-cigarettes on oral health according to education/professional training.

	Dentist		Specialist		*p*-Value
	N	%	N	%	
Total	103	100.0	86	100.0	
**Do e-cigarettes affect the increase in the incidence of caries?**					
Yes	56	54.4	38	44.2	*p* = 0.361
No	11	10.7	10	11.6	
I don’t know	36	35.0	38	44.2	
**Does the use of e-cigarettes affect the oral cavity?**					
Yes	79	76.7	63	73.3	*p* = 0.290
No	1	1.0	4	4.7	
I don’t know	23	22.3	19	22.1	
**Do e-cigarettes affect xerostomia, mouth burning, and halitosis?**					
Yes	74	71.8	59	68.6	*p* = 0.745
No	-	-	3	3.5	
I don’t know	29	28.2	24	27.9	
**Do e-cigarettes increase the risk of oral cancer (cytotoxic, genotoxic, and oncogenic effects)?**					
Yes	70	68.0	55	64.0	*p* = 0.670
No	-	-	1	1.2	
I don’t know	33	32.0	30	34.9	
**Do e-cigarettes affect the prevalence of periodontal lesions?**					
Yes	66	64.1	56	65.1	*p* = 0.594
No	10	9.7	5	5.8	
I don’t know	27	26.2	25	29.1	
**Do e-cigarettes affect lingua nigra villosa, nicotinic stomatitis, and other tongue discolorations?**					
Yes	67	65.0	49	57.0	*p* = 0.375
No	2	1.9	4	4.7	
I don’t know	34	33.0	33	38.4	
**Do e-cigarettes affect teeth (tooth discoloration, pain, fractures, and increase in cariogenic bacteria)?**					
Yes	69	67.0	52	60.5	*p* = 0.618
No	7	6.8	6	7.0	
I don’t know	27	26.2	28	32.6	
**Do e-cigarettes affect disorders of the oral microbiome?**					
Yes	72	69.9	51	59.3	*p* = 0.266
No	3	2.9	5	5.8	
I don’t know	28	27.2	30	34.9	

The statistical test used for comparisons in this table was the Chi-square test (χ^2^). No significant differences were found between dentists and specialists for any of the categories presented (*p* > 0.05 for all comparisons).

## Data Availability

The data presented in this study are available on request from the corresponding authors.
